# Penicillin-Binding Protein-4 (PBP4) of *Staphylococcus aureus* and Its Role in β-Lactam Resistance: An Update

**DOI:** 10.3390/microorganisms14040917

**Published:** 2026-04-18

**Authors:** Nidhi Satishkumar, Som S. Chatterjee

**Affiliations:** 1 Department of Microbial Pathogenesis, School of Dentistry, University of Maryland, Baltimore, MD 21201, USA; 2 Institute of Marine and Environmental Technology (IMET), Baltimore, MD 21202, USA

**Keywords:** *S. aureus*, Penicillin-binding protein-4 (PBP4), β-lactam resistance

## Abstract

*Staphylococcus aureus* remains to be one of the leading causes of global mortality. The most common class of antibiotics used to treat *S. aureus* infections are next-generation β-lactams (NGBs), as they are highly efficacious and have low adverse effects. NGB resistance in *S. aureus* is classically attributed to penicillin-binding protein-2a (PBP2a), but previous studies from our group have also implicated an altered expression of penicillin-binding protein-4 (PBP4) with NGB resistance. PBP4 is the sole low-molecular-mass (LMM) PBP present in *S. aureus*; it is also the only known LMM PBP with transpeptidase activity, giving it the unique ability to bring about peptidoglycan cross-linking. In this article, we review some of the recent findings from our group, which reveal that mutations associated with PBP4 lead to altered protein expression and NGB resistance in both methicillin-susceptible *S. aureus* (MSSA) and methicillin-resistant *S. aureus* (MRSA) backgrounds. We discuss the clinical relevance of PBP4-associated mutations, particularly in methicillin-resistant lacking *mec* (MRLM) isolates, as well as the synergistic effect of altered PBP4 and GdpP functions. Finally, this review summarizes the potential role played by PBP4 in *S. aureus* virulence. Together, we highlight the increasing relevance of PBP4 as a mediator of NGB resistance and discuss its potential as an important factor during infection diagnosis and therapy.

## 1. Introduction

*Staphylococcus aureus*, the Gram-positive opportunistic human pathogen, can cause skin infections, bacteremia, and sepsis (among other infections). The bacterium is also well-known for causing persistent or relapsing infections due to its ability to evade the action of antibiotic treatment through mechanisms of drug resistance or tolerance, and has a significant impact on global health and economy. In 2019 alone, infections caused by *S. aureus* resulted in over a million global deaths in individuals older than 15 years. *S. aureus* was the leading bacterial pathogen that resulted in death in 135 countries and caused a YLL (years of life lost) burden of 34.3 million. Moreover, *S. aureus* was the leading cause of mortality associated with bloodstream infections, resulting in 299,000 global deaths in 2019 [1,2]. The treatment of *S. aureus* infections is largely dependent on antibiotics that target vital cellular processes and inhibit bacterial growth and survival. However, the bacterium is infamous for developing resistance towards virtually all available antibiotics, rendering them ineffective [3]. The development of vaccines targeting *S. aureus* has been consistently unsuccessful due to a plethora of virulence factors, high genetic variability among strains, and the range of diseases caused by the bacterium [4]. The highly pathogenic nature of the bacteria, combined with their ability to evade antibiotic treatment and the lack of an efficient vaccine, makes *S. aureus* a successful modern pathogen.

Today, the most common classes of antibiotic drugs used to treat *S. aureus* infections include β-lactams, glycopeptides, cyclic lipopeptides, and oxazolidones [5]. β-lactams, in particular, are deemed largely successful due to their high efficacy, superior tissue distribution, and low adverse effects compared to the other drug classes, and they account for 65% of all injectable antibiotics that are prescribed in the United States [6]. They inhibit the process of bacterial cell wall assembly by targeting penicillin-binding proteins (PBPs), which are enzymes that perform peptidoglycan cross-linking, thereby causing cell lysis and death [7]. Given its clinical relevance, our group’s research has focused on elucidating mechanisms of resistance to next-generation β-lactams (NGBs). In our earlier studies, we demonstrated the potential of penicillin-binding protein-4 (PBP4) as an important mediator of NGB resistance and summarized its relevance in a review in 2018 [8]. Since then, research conducted by us and other groups on this topic has further discussed the relevance of PBP4 in NGB resistance, virulence, and its potential as a target for therapy development. This review summarizes these recent developments and serves as an updated version of our previous article.

## 2. Peptidoglycan

**Figure 1:**
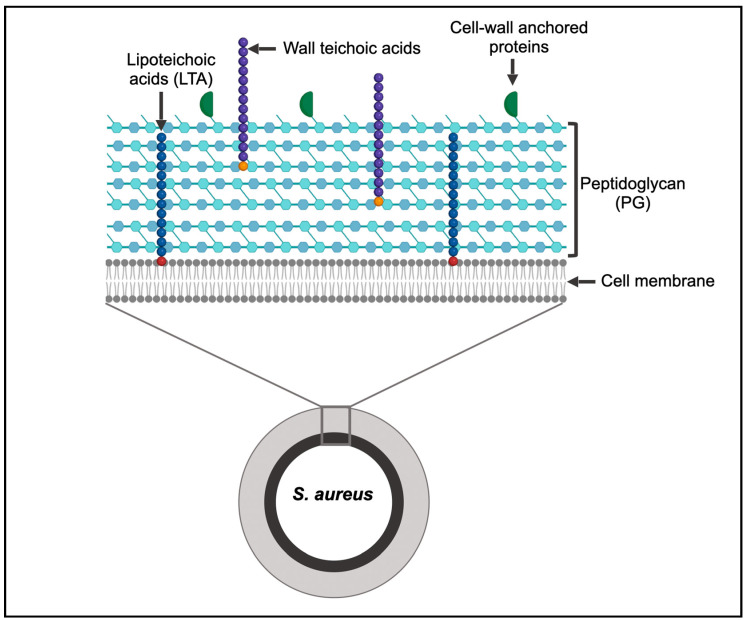
Structure and composition of the *S. aureus* cell wall. The cell wall is a continuous, outermost layer of the cell and is composed of polymers, including the peptidoglycan and teichoic acids, in addition to surface-anchored proteins. Created in BioRender. Satishkumar, N. (2026) https://BioRender.com/5asbaju.

The bacterial cell wall, a continuous outer layer of the cell, is an essential feature, as it provides shape, structure, and protection from external stressors [9] (Figure 1). Almost all bacterial species possess a cell wall, with the exception of mycoplasma and L-form bacteria [10]. The cell wall does not only serve as a physical barrier from the environment but also plays important roles in cell metabolism, cell division, pathogenesis, and antibiotic resistance, which makes it an attractive target of antibacterial agents [11]. It is a complex structure mainly consisting of sugars, lipids, and protein molecules, the composition of which varies among Gram-positive and Gram-negative bacteria. The cell wall of Gram-positive bacteria comprises a thick layer of peptidoglycan along with teichoic acid polymers and surface proteins [12] (Figure 1). Gram-negative bacteria also possess peptidoglycan, but it is relatively thin, and it is present between the inner and the outer cell membrane, the latter of which has a lipopolysaccharide layer (LPS) anchored to it.

**Figure 2:**
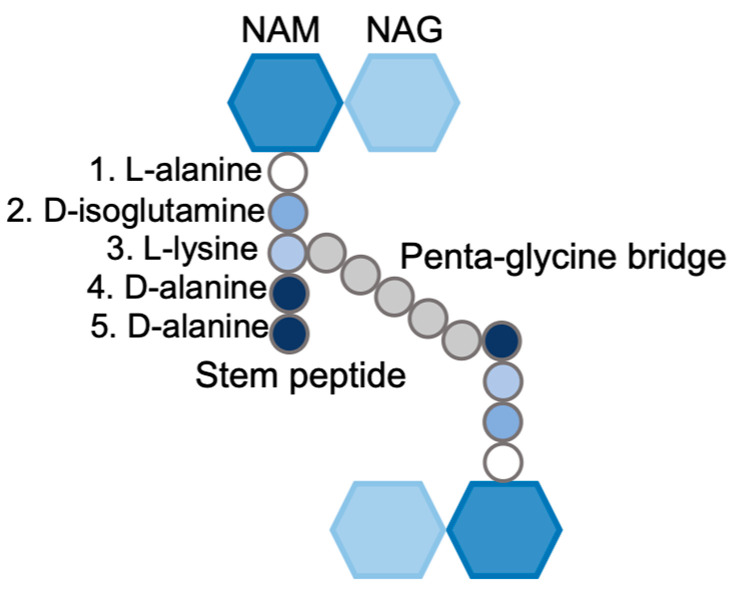
Components of the peptidoglycan monomer in *S. aureus*.

**Figure 3:**
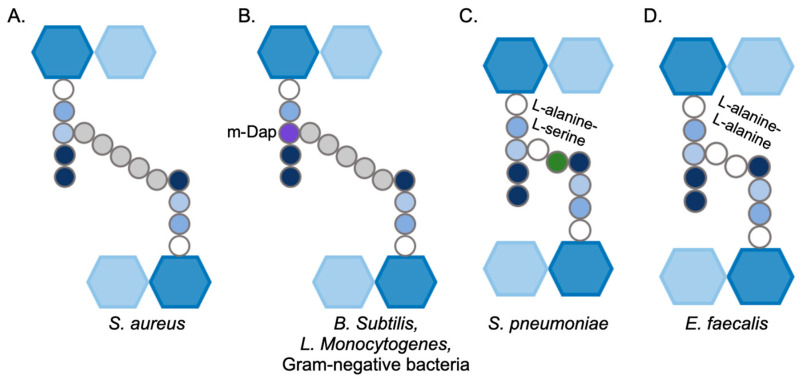
Variations in composition of peptidoglycan monomers among bacterial species. (**A**) *S. aureus*; (**B**) *B. subtilis*, *L. monocytogenes* and Gram-negative bacteria; (**C**) *S. pneumoniae*; (**D**) *E. faecalis*.

**Figure 4:**
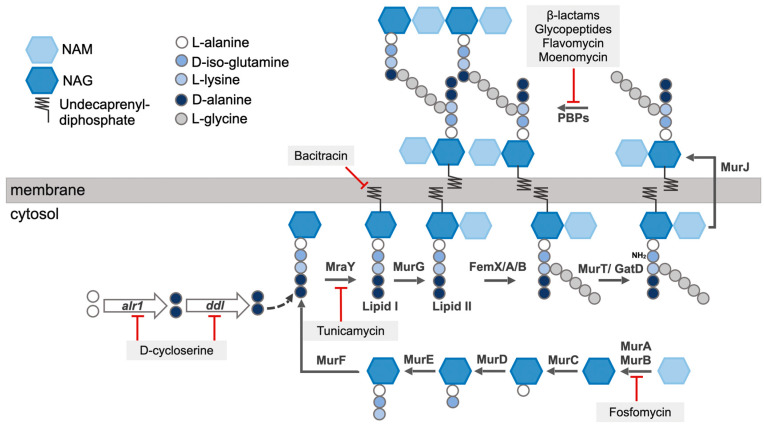
*S. aureus* peptidoglycan synthesis begins in the cell cytosol, following which the monomer is assembled and anchored onto the cell membrane (lipid II). Lipid II is flipped to the extracellular space, where it is cross-linked by PBPs. Antibiotics targeting the peptidoglycan synthesis process are indicated.

The structure, shape, and integrity of the cell are attributed to the peptidoglycan, a continuous polymer spanning the cell surface. The peptidoglycan is a scaffolding structure composed of glycan polymers interconnected through peptide chains [12]. In Gram-positive bacteria, the peptidoglycan is present in multiple layers, and it is approximately 20–40 nm thick. Gram-negative bacteria have one or a few layers of peptidoglycan, and they range from 1.5 to 10 nm in thickness [13]. Peptidoglycan is also dynamic in nature; the thickness of the structure varies based on the stage of cell division, cell growth, and pathogenesis [14]. The single repeating unit of the peptidoglycan, a monomer, comprises a disaccharide unit, a stem peptide, and a bridge peptide. The disaccharide is composed of N-acetylmuramic acid (NAM) and N-acetylglucosamine (NAG), and the stem peptide is associated with NAM at the D-lactyl site [15,16]. This stem peptide comprises five amino acids: L-alanine, D-iso-glutamine, L-lysine, D-alanine, and D-alanine [9] (Figure 2). The NAM-NAG disaccharide and the first amino acid of the stem peptide, L-alanine, are largely conserved in the majority of bacterial species. In certain Gram-positive bacteria, such as *Bacillus subtilis* [17] and *Listeria monocytogenes*, and in the majority of Gram-negative species, the third position of the stem peptide consists of *meso*-diaminopimelic acid (*m*-DAP) instead of L-lysine [18] (Figure 3). Notably, the D-alanine-D-alanine amino acids at the stem peptide terminus are highly conserved, a feature that is exploited by peptidoglycan-targeting antibiotics such as glycopeptides [19] (Figure 4). The third position of the stem peptide comprises a bridge peptide, the composition of which varies among bacterial species [16]. In *S. aureus*, the bridge peptide comprises five units of glycine and is referred to as the pentaglycine cross-bridge [9]. The bridge peptide in *Streptococcus pneumoniae* [20] and *Enterococcus faecalis* [21] is relatively shorter and is composed of L-alanine-L-serine, and L-alanine-L-alanine, respectively (Figure 3). Linear polymerization of NAM-NAG units is formed via a glycosidic bond among adjacent disaccharides, whereas cross-linking occurs via the formation of an amide bond between the terminal amino acid of the bridge peptide and D-alanine, the fourth amino acid of the stem peptide of an adjacent disaccharide [9]. The average length of the glycan chains and the extent of peptidoglycan cross-linking vary among species. In *S. aureus*, the average peptidoglycan chain length is typically shorter, comprising of around six monomers [22]. However, 80–90% of the stem peptides in *S. aureus* are cross-linked [23]. *B. subtilis* has longer peptidoglycan chains of around 50–250 monomers, but only 40% of the stem peptides are cross-linked [17]. *E. coli* strains have a large variety in peptidoglycan chain lengths, ranging from one to 30 monomers, but only 30% of them are typically cross-linked [24]. The presence of both short and long glycan chain lengths suggests that chain length likely does not determine peptidoglycan thickness. However, in certain bacteria, peptidoglycan cross-linking plays an important role in cell morphology. In *S. aureus*, the cross-linking of stem peptides is essential for peptidoglycan integrity and cell survival [25]. On the other hand, the relaxation of peptidoglycan cross-linking enables *H. pylori* cells to maintain a curve in their shape that is vital for successful colonization [26]. Linear polymerization and cross-linking of adjacent peptidoglycan disaccharides are both performed by enzymes collectively called penicillin-binding proteins (PBPs) [27].

### Peptidoglycan Synthesis

The synthesis of the *S. aureus* peptidoglycan begins intracellularly with the assembly of a membrane-anchored monomer, which is subsequently flipped across the cell membrane and is assimilated into the growing peptidoglycan in the extracellular space [28] (Figure 4). In *S. aureus*, NAG is first synthesized from glucosamine-6-phosphate by GlmM and GlmU in the cytosol, which is then converted to NAM by MurA and MurB enzymes [29]. The Mur ligases, MurC, MurD, MurE, and MurF, further bring about the sequential addition of L-alanine, D-iso-glutamic acid, L-lysine, and D-alanyl-D-alanine to NAM, respectively, completing the assembly of the stem peptide [30]. The terminal D-alanyl-D-alanine dipeptide is synthesized from two L-alanine molecules via the enzymatic activity of proteins encoded by *alr1* and *ddl*. Alr1 (alanine racemase) mediates the conversion of L-alanine to D-alanine, while Ddl (alanine ligase) mediates the formation of D-alanyl-D-alanine dipeptide [31]. The assembly of the peptidoglycan monomer is an attractive antibiotic target, as causing its inhibition leads to stalling of the peptidoglycan synthesis process at the cytosolic stage. The antibiotic fosfomycin [32] binds to the active site of MurA, causing the enzyme to be inactivated. Similarly, D-cycloserine inhibits Alr1 and Ddl, preventing the formation of the D-alanyl-D-alanine dipeptide [31]. Next, MraY, a membrane translocase, links the assembled precursor unit to undecaprenyl diphosphate, generating lipid I. The formation of lipid I is stalled by the antibiotic tunicamycin, which inhibits MraY [33]. MurG catalyzes the generation of lipid II by adding NAG to lipid I precursor. The addition of the pentaglycine bridge to lipid II is catalyzed by the enzymes FemX/A/B, completing the synthesis of a peptidoglycan monomer [25]. Finally, D-iso-glutamic acid, the second amino acid of the stem peptide, is converted to D-iso-glutamine by amidation performed by MurT/GatD [34,35]. A flippase enzyme, MurJ, then flips the assembled lipid II toward the membrane outer leaflet [36]. In the extracellular region, lipid II-linked monomers are polymerized and cross-linked by the transglycosylation and transpeptidation activities of PBPs [37]. The nascent, lipid-linked peptidoglycan chains are released from the lipid carrier by the SagB-SpdC complex and are subsequently incorporated into the pre-existing peptidoglycan [38], while the lipid carrier is recycled to continue with the transport of more peptidoglycan monomers [39]. The processing of lipid II is an important target of various antibiotics. β-lactams such as nafcillin or oxacillin inhibit the transpeptidation activity of the proteins, while moenomycin [40] or flavomycin inhibit transglycosylation. Glycopeptides like vancomycin or teicoplanin specifically bind to the D-alanyl-D-alanine stem peptide of lipid II-linked peptidoglycan to inhibit PBP-mediated transpeptidation [19]. Lysostaphin, a metalloprotease bacteriocin synthesized by *S. simulans* specifically cleaves the bridge peptide between the third and fourth glycine residues [41]. Bacitracin, a bacteriocin produced by *Bacillus licheniformis*, blocks the recycling of lipid carriers [42].

The peptidoglycan synthesis and cross-linking machinery is concentrated at the septum, the division site of replicating bacteria, while the mature, pre-existing peptidoglycan is dispersed toward the peripheral region of the cell. The process of peptidoglycan synthesis is highly regulated and works in coordination with enzymes of the cell division machinery, such as FtsZ, GpsB, or DivIC, and cell wall hydrolases, such as Atl, SagB, LytN or LytM [43]. PBPs and cell wall hydrolases work in concert to maintain a balance in peptidoglycan synthesis and hydrolysis, ensuring cell viability.

## 3. Penicillin-Binding Proteins (PBPs)

**Figure 5:**
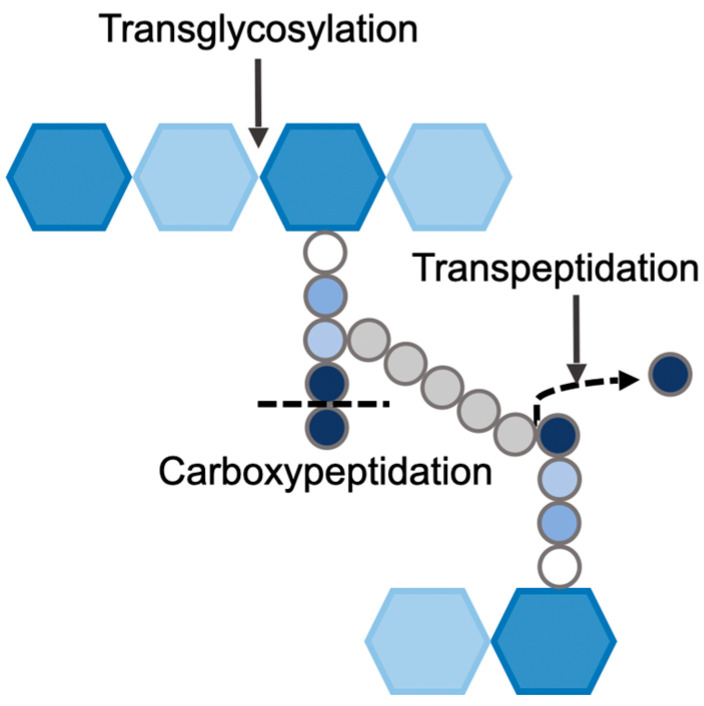
Functions performed by PBPs in *S. aureus*.

The substrates of penicillin were found to be a group of enzymes that perform cross-linking of bacterial peptidoglycan. These enzymes, due to their affinity towards penicillin, were termed penicillin-binding proteins (PBPs) [44]. PBPs are membrane-anchored proteins found in all bacterial species. They are multi-domain structures, each with an associated function [45]. They can polymerize and/or cross-link the peptidoglycan monomers through transglycosylation [40] (the formation of a glycosidic bond between adjacent NAM-NAG disaccharides) and transpeptidation [7] (the formation of the cross-bridge between adjacent peptidoglycan stem peptides) activities, respectively (Figure 5). Certain PBPs also have carboxypeptidation (hydrolysis of the terminal D-alanine of stem peptide) or endopeptidation (hydrolysis of the bridge peptide) activities. PBPs, in addition to maintaining the shape and thickness of the peptidoglycan, also control the extent of peptidoglycan cross-linking. Each PBP has one or two of the above-mentioned functions, and bacteria often possess multiple PBPs.

### 3.1. Classification of PBPs

Both Gram-positive and Gram-negative bacteria possess PBPs, which are primarily categorized based on their molecular mass. The molecular mass of each PBP also dictates its nomenclature, as they are annotated based on their pattern of separation following SDS-PAGE [37]. PBPs are broadly categorized into three classes on the basis of their domains and associated functions.

**Figure 6:**
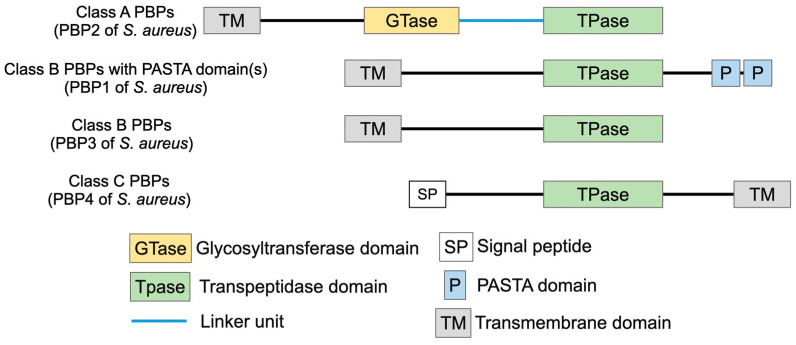
PBPs are composed of multiple domains with different functions.

Class A PBPs are high-molecular-mass (HMM) PBPs and contain both the glycosyltransferase domain and the transpeptidase domain. They are bi-functional and bring about polymerization of the glycan chain (via the glycosyltransferase domain) in addition to the cross-linking of stem peptides (via the transpeptidase domain). The glycosyltransferase domain associated with class A PBPs is present at the N-terminal region (Figure 6). The general structure of this domain is conserved among bacterial species, with five highly conserved amino acid motifs surrounding two catalytically active glutamic acid residues [40]. They are separated from the transpeptidase domain by a spacer to allow both transglycosylation and transpeptidation activities simultaneously. The transglycosylation activity is exclusive to class A PBPs, making them essential PBPs.

Class B PBPs are monofunctional HMM proteins, and they only have transpeptidation function. The transpeptidase domains of class A and class B PBPs are structurally similar, with the consistent characteristic of a penicillin-binding (PB) domain. The PB domain includes the transpeptidation active site (SXXK motif) and its associated conserved motifs (SXN and the KTG motifs) [46,47]. Transpeptidation begins with the binding of PBPs to the peptidoglycan stem peptide, where the serine of the SXXK active site motif attacks the terminal D-alanyl-D-alanine bond, forms an acyl–enzyme complex with the stem peptide, and concomitantly releases the D-alanine from the terminal end. The C-terminal peptidyl moiety is subsequently transferred onto the terminal glycine of the bridge peptide of the neighboring peptidoglycan monomer, completing the bridge formation [45]. The SXN motif is associated with substrate binding and deacetylation following transpeptidation, whereas lysine of the KTG motif has been associated with the activation of the SXN motif [48]. β-lactams, being structurally analogous to the D-alanyl-D-alanine dipeptide, also bind to the SXXK motif. The antibiotics first form a non-covalent Michaelis complex with the PB domain region, following which the serine of the active site launches a nucleophile attack on the β-lactam ring to form an acyl–enzyme complex, which inhibits further transpeptidase activity [49].

Class C PBPs are low-molecular-mass (LMM) proteins that have peptidoglycan hydrolase activity either in the form of carboxypeptidation or endopeptidation. Carboxypeptidation and endopeptidation activities bring about a decrease in the extent of peptidoglycan cross-linking. Due to this, they play vital roles in peptidoglycan remodeling and recycling [45]. LMM PBPs also have a PB domain associated with them, including the transpeptidase domain with the conserved SXXK, SXN, and KTG motifs. This allows LMM PBPs to recognize and bind the D-alanyl-D-alanine stem peptide. However, while they can attack the stem peptide and form an acyl–enzyme complex, they are unable to accept a recipient amino acid to transfer the peptidyl moiety, thus resulting in a shorter stem peptide and a released D-alanine. PBP4 of *S. aureus* is an exception, as it is the only known LMM PBP with transpeptidation activity.

### 3.2. PBPs of S. aureus

*S. aureus* naturally contains four PBPs—PBP1 through to PBP4. The PBP with the highest molecular mass, PBP1, is a class B monofunctional transpeptidase. PBP1 deletion leads to abnormal septal formation, suggesting that PBP1, in addition to cross-linking the peptidoglycan, is also part of the cell division machinery [50,51]. In addition to the transpeptidase domain, the C-terminal of PBP1 consists of two PASTA (penicillin-binding protein and serine/threonine kinase-associated) domains. The PASTA domains of PBP1 are essential for cell growth and protein functionality and are shown to bind to uncross-linked peptidoglycan [51]. PBP2 belongs to class A and has the ability to perform both transglycosylation and transpeptidation activities. It is the sole class A PBP of *S. aureus*, due to which it is considered essential for bacterial growth and survival [52]. PBP3, a class B PBP, is associated with the cross-linking of the peripheral peptidoglycan, in addition to mediating cell elongation during cell division via its association with proteins of the cell divisome such as RodA [53,54]. PBP4 is the only low-molecular-mass (LMM) PBP of *S. aureus*. LMM PBPs belong to class C and are characterized by their carboxypeptidase and/or endopeptidase activities. However, PBP4 of *S. aureus* has transpeptidation activity in addition to carboxypeptidation, making it the only known LMM PBP to be able to perform peptidoglycan cross-linking [55,56,57].

### 3.3. Penicillin-Binding Protein-2a (PBP2a)

Resistance to penicillin, the first β-lactam used for the treatment of bacterial infections was detected soon after the drug was introduced clinically to treat infections [58]. Penicillin resistance is attributed to a penicillinase, now known as β-lactamase. The *blaZ*-encoded β-lactamase hydrolyzes and inactivates the β-lactam ring of penicillin and renders it inactive [59]. Subsequent years witnessed the development of methicillin, a β-lactam used to treat infections caused by strains resistant to penicillin. In 1961, strains resistant to methicillin were detected, marking the surge of methicillin-resistant *S. aureus* (MRSA). In MRSA, β-lactam resistance is attributed to PBP2a, a horizontally acquired PBP with low affinity towards the drug, which leads to decreased drug binding and results in resistance to methicillin [60]. PBP2a is encoded by *mecA*, which is part of the mobile genetic element Staphylococcal cassette chromosome-*mec* (SCC-*mec*) and confers broad-spectrum resistance to subsequently introduced types of β-lactams such as cephalosporins and carbapenems. While the three transpeptidase motifs of PBP2a remain conserved, kinetic studies revealed that the acyl–enzyme complex formation between PBP2a and β-lactams occurs insufficiently, allowing β-lactam resistance to occur [61]. In 2019, MRSA led to over 100,000 global deaths associated with drug resistance, demonstrating its significance as a pathogen responsible for a high health and economic burden over 50 years after it was initially identified [2].

Strains lacking *mecA* (or, *mecC* [62], a homolog of *mecA*) are classified as MSSA (methicillin-susceptible *S. aureus*), with narrow-spectrum resistance to early-generation β-lactams such as penicillin. MSSA strains are treated with NGBs such as nafcillin or oxacillin [63]. Infections caused by MRSA, identified by the presence of *mecA*, are treated with advanced-generation cephalosporins, such as ceftaroline (or ceftabiprole), which are designed to specifically bind and inhibit PBP2a [64]. Recent years have seen the detection of ceftaroline-resistant MRSA owing to structural alterations in PBP2a that lead to decreased affinity towards ceftaroline [65]. Ceftaroline resistance warrants the use of antibiotics belonging to other drug classes such as vancomycin or daptomycin. However, these alternative classes of antibiotics, albeit effective, have significant adverse effects, such as nephrotoxicity, and pose the risk of infection relapse [66].

### 3.4. PBP4

**Figure 7:**
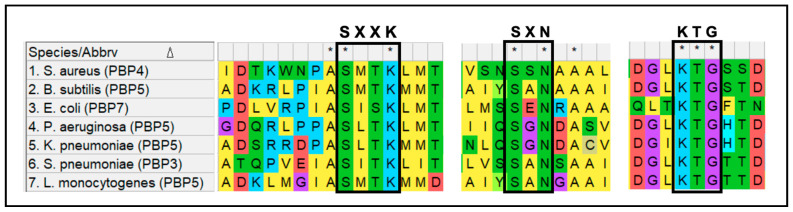
Multiple amino acid sequence alignment of the conserved SXXK, SXN and KTG motifs of LMM PBPs of *S. aureus* (PBP4), *B. subtilis* (PBP5), *E. coli* (PBP7), *P. aeruginosa* (PBP5), *K. pneumoniae* (PBP5), *S. pneumoniae* (PBP3) and *L. monocytogenes* (PBP5). Asterisks (*) represents conserved amino acids of above mentioned motifs.

PBP4 of *S. aureus* has transpeptidation activity in addition to carboxypeptidation, making it the only known LMM PBP to be able to perform peptidoglycan cross-linking [55,56,57]. Studies revealed the ability of PBP4 to add amino acids onto not only peptidoglycan polymers, but also lipid I and lipid II, suggesting its propensity towards transpeptidation over carboxypeptidation [67]. Multiple sequence alignment of PBP4 from *S. aureus* and LMM PBPs from other bacterial species, such as *B. subtilis* (PBP5), *E. coli* (PBP7), *Listeria monocytogenes* (PBP5), *P. aeruginosa* (PBP5), *K. pneumoniae* (PBP5), and *S. pneumoniae* (PBP3), revealed that the key motifs associated with the active site, SXXK, SXN, and KTG remained highly conserved in PBP4 (Figure 7). While this hinted toward the possibility that the transpeptidation activity of PBP4 was likely not attributed to potential alterations in the active site, further confirmation by structural and biochemical investigations is required. Thus, the exact mechanism by which PBP4 of *S. aureus* can perform peptidoglycan cross-linking remains unknown. The conditions that dictate *S. aureus* PBP4 to perform either carboxypeptidase or transpeptidase activity are also unknown. However, the deletion or inactivation of PBP4 from *S. aureus* led to a significant decrease in cell wall cross-linking, suggesting its propensity toward transpeptidation [68].

## 4. Non-Classical Mechanisms of NGB Resistance in *S. aureus*

### 4.1. pbp4-Associated Mutations Lead to β-Lactam Resistance in S. aureus

Recent years have seen a rise in the detection of *mecA*-negative, NGB-resistant isolates of *S. aureus*, potentially suggesting the presence of certain non-classical NGB resistance mechanisms [69,70,71]. This increasing detection of *mecA-*negative, NGB-resistant *S. aureus*, combined with the ability of the pathogen to rapidly gain new resistance mechanisms, prompted studies that explored potentially novel mechanisms of β-lactam resistance that did not depend on *mecA*. To that end, our early studies involved passaging of *S. aureus* in increasing concentrations of the advanced-generation β-lactam, ceftobiprole, until the bacteria gained the ability to survive in the presence of the drug [72,73]. The parent, susceptible strain used for the study, was COLnex, the *mecA*and *blaZ* (the classical mediators of β-lactam resistance)lacking variant of COL, which is a prominent, archaic MRSA strain (Table 1). Serial passaging of COLnex in sub-inhibitory concentrations of ceftobiprole for 21 days resulted in the generation of CRB, a resistant variant. The minimum inhibitory concentration (MIC) of CRB was 128 mg/L. This MIC for CRB was 512-fold more than that for COLnex, which has an MIC of less than 0.25 mg/L for ceftobiprole. To identify the potential genetic alterations that led to the resistant phenotype in CRB, whole genome sequencing was carried out with COLnex and CRB. CRB was found to contain mutations associated with two specific genes— *pbp4* and *gdpP*. *-* *pbp4* had two missense mutations (E183A and F241R) associated with it, in addition to a 36 bp duplication located 290 bp upstream of the *pbp4* start codon, in the regulatory region. *gdpP* contained a missense mutation (N182K) in its coding region (Table 1) [74]. Of the two candidate genes, *pbp4* has been previously associated with low-level β-lactam resistance [75]. More importantly, the deletion of *pbp4* in CRB led to a significant decrease in MIC to ceftobiprole (MIC < 0.25 mg/L), such that it was comparable with the parent strain, COLnex, suggesting that PBP4, which usually does not play a very prominent role in NGB resistance, was likely responsible for the resistant phenotype seen in CRB [76].

Following the indication that alterations associated with PBP4 expression potentially led to resistance to β-lactams, studies were carried out to determine the specific phenotypes attributed to the *pbp4*-associated mutations in CRB. The regulatory-site-associated mutation and the missense mutations were each individually introduced into COLnex, resulting in COLnex Ppbp4* (CRB) and COLnex pbp4** (CRB), respectively (Table 1). Since the Ppbp4* (CRB) mutation was located in the regulatory region, its effect on protein expression was first analyzed. Bocillin-Fl binding assay detected significantly increased levels of PBP4 in COLnex Ppbp4* (CRB) compared to COLnex wild-type, suggesting that the mutation led to PBP4 overexpression due to potential alterations in the regulation of protein expression [76]. As PBP4 is involved in cell wall cross-linking, the peptidoglycan from COLnex wild-type and COLnex Ppbp4* (CRB) were purified and muropeptide composition was analyzed by HPLC. COLnex Ppbp4* (CRB) had significantly enhanced peptidoglycan cross-linking compared to COLnex wild-type, as witnessed by an increase in the amount of long-chained oligomeric units or the “hump” and a corresponding decrease in short-chained muropeptides, such as monomers and dimers. COLnex Ppbp4* (CRB) was also highly resistant to NGBs, with a reported 16-fold increase in MIC for nafcillin, 128-fold increase in MIC for ceftaroline, and a four-fold increase in MIC for ceftobiprole when compared to COLnex [76]. Taken together, this study revealed that the regulatory-site-associated mutation in CRB resulted in the overexpression of PBP4, subsequently leading to increased peptidoglycan cross-linking, which resulted in NGB resistance. This mechanism of NGB resistance was novel, as hitherto known resistance mechanisms were dependent either on drug inactivation (as displayed by the β-lactamase PC1, mediated by *blaZ*), or on reduced drug affinity (as displayed by the expression of PBP2a, mediated by *mecA*) [3], and thus warranted further investigation.

In order to assess the effect of the missense mutations associated with *pbp4*, the gene from either wild-type bacteria (*pbp4* wt), or from CRB (pbp4**) was introduced into an isogenic, heterologous host and challenged with β-lactam treatment. Variants of *pbp4* were introduced into COLnex Δ*pbp4* mutant via the constitutively expressing plasmid, *pTX*_Δ_, and a population assay was performed with nafcillin. The complementation with pbp4** resulted in a significantly higher number of colony-forming units (measured as CFU/mL) in the presence of nafcillin when compared to the complementation with *pbp4* wt, suggesting that the pbp4** missense mutations led to NGB resistance. [77]. The introduction of both the regulatory-site-associated and missense mutations into COLnex, namely COLnex Ppbp4* (CRB) pbp4** (CRB), also led to an increased level of NGB resistance (as indicated by significantly higher amounts of CFU/mL in presence of nafcillin) compared to that indicated by either COLnex Ppbp4* (CRB) or COLnex pbp4** (CRB) [78]. Subsequent studies were performed by serially passaging NGB-susceptible strains in other antibiotics such as nafcillin or ceftaroline. Similar to that seen in CRB, the resultant variants were significantly more resistant to NGBs compared to their respective parent strains, and had accumulated mutations associated with the *pbp4* gene [77]. Taken together, these studies demonstrated that PBP4 can play an important role in non-classical NGB resistance through altered expression (mediated by regulatory-site-associated mutations) and/or function (mediated by missense mutations).

### 4.2. Relevance of pbp4-Associated Mutations in Clinically Isolated Strains

On determining that the *pbp4* regulatory-site-associated and missense mutations played an important role in NGB resistance in laboratory-passaged strains, our group conducted studies to determine if the mutations and their associated phenotypes were also relevant in clinically isolated strains of *S. aureus*. Findings of a retrospective study conducted in Belgium revealed the presence of clinical isolates of *S. aureus* that were phenotypically resistant to β-lactams and were devoid of *mec* genes. These strains, termed either MRLM (methicillin-resistant lacking *mec*) or BORSA (borderline-oxacillin resistant *S. aureus*), contained mutations associated with *pbp1*, *pbp2*, *pbp3*, *pbp4* (including its regulatory region), *yjbH*, and *gdpP* [69]. To determine if the mutations detected in clinical strains had similar phenotypic effects as those displayed by the laboratory-generated strains discussed above, our group assessed the effect of representative *pbp4-*associated mutations by introducing them into isogenic, heterologous host bacterial cells. The effect of five representative regulatory-site-associated mutations (Ppbp4*) were assessed via a luciferase reporter assay. The findings of this study revealed that three of the five mutation types had significantly increased promoter activity when compared to the wild-type promoter, suggesting that these mutations potentially led to increased PBP4 expression [79]. Indeed, the introduction of one of these mutations (T to A substitution 266 bp upstream of the *pbp4* start codon) into the genome of COLnex, generating the strain COLnex Ppbp4* (Strain 1), led to PBP4 overexpression when compared to COLnex wild-type. Moreover, growth curve analysis indicated that, compared to COLnex wild-type, COLnex Ppbp4* (Strain 1) had increased survival when exposed to nafcillin or cefoxitin, suggesting its ability to be resistant to NGBs.

Similarly, the effect of missense mutations associated with the *pbp4* gene was assessed by introducing a representative mutation, R200L, into the genome of COLnex, generating COLnex pbp4** (R200L). On treatment with β-lactams, COLnex pbp4** (R200L) had significantly increased growth when compared to COLnex wild-type, suggesting that the R200L mutation played an important role in NGB resistance. Interestingly, the R200L mutation was able to mediate increased NGB resistance (as seen by increased survival in the presence of nafcillin or cefoxitin) compared to other missense mutations studied, including those seen in CRB, suggesting that each missense mutation potentially had unique mechanisms of resistance [79]. These studies confirmed that *pbp4-*associated mutations are not only prominent in clinically isolated, β-lactam-resistant strains of *S. aureus*, but that they also play a role in contributing toward the resistant phenotype. *pbp4*-associated mutations have since been detected in at least two other surveillance studies [70,71]. An analysis to determine the relevance of these mutations revealed that of a total of 194 MRLM isolates detected, 84% (163 isolates) contained at least one type of *pbp4*-associated mutation [80], signifying the global prevalence of these mutations and the potential impact of PBP4-mediated NGB resistance.

The findings of the above-discussed studies indicated that *S. aureus* strains with regulatory-site-associated *pbp4* mutations are significantly more resistant to NGBs when compared to strains consisting of missense mutations, suggesting that PBP4 overexpression and increased peptidoglycan cross-linking were vital for NGB resistance. Further studies were therefore focused on the regulatory-site-associated mutations. While the phenotypes associated with the regulatory-site-associated *pbp4* mutations were well-described in the MSSA background, as discussed above, [77], the role of the mutations in MRSA strains was unknown. To determine this, three different types of representative regulatory-site-associated mutations were introduced into SF8300, a derivative of USA300, a prominent community-associated MRSA (CA-MRSA) detected in the United States [81]. An immunoblotting assay demonstrated that, consistent with previous findings, the introduction of regulatory-site-associated mutations led to PBP4 overexpression in SF8300, too. Growth studies indicated that SF8300 containing regulatory-site-associated mutations also had increased survival in the presence of NGBs when compared to SF8300 wild-type [82]. Moreover, the increase in resistance to NGBs corresponded to the levels of PBP4 overexpression displayed by the mutants, reiterating that PBP4 overexpression was the cause of NGB resistance. These findings suggested that PBP4 overexpression supplemented PBP2a-associated resistance seen in MRSA strains and that infections caused by MRSA strains containing PBP4-associated mutations were potentially more challenging to treat.

The *pbp4* gene shares its regulatory region (P*pbp4*) with *abcA*, a gene that encodes for an ATP-binding cassette (ABC) transporter protein. ABC transporters are known for mediating resistance to anti-infectives in prokaryotes, as well as eukaryotes, via export activity [83,84]. As AbcA has been associated with antibiotic resistance, it was necessary to determine whether the regulatory-site-associated mutations caused alterations in the expression of *abcA* transcripts, which potentially led to AbcA-mediated NGB resistance. qRT-PCR, used to determine the effect of regulatory-site-associated mutations, indicated that while the presence of mutations in P*pbp4* led to increased levels of *pbp4* transcripts, they also led to a corresponding decrease in levels of *abcA* transcripts. These findings suggested that the regulatory-site-associated mutations potentially led to decreased AbcA expression and that the resistant phenotype was likely attributed to PBP4 overexpression.

## 5. Auxiliary Effects of *pbp4*-Associated Mutations

### 5.1. Synergistic Effect of pbp4-Associated Mutations and gdpP Alterations

While *pbp4*-associated mutations did result in NGB resistance, the increase in resistance was not as substantial as that reported in CRB, as CRB was 512-fold more resistant than COLnex to nafcillin, whereas COLnex Ppbp4* (CRB) pbp4** (CRB) was only 16-fold more resistant than COLnex [78]. While CRB did not contain mutations associated with any other *pbp*s, it did contain mutations associated with GdpP, a phosphodiesterase that cleaves the second messenger cyclic-di-AMP (CDA). CDA plays an important role in bacterial cell physiology and metabolism [73,85]. The *gdpP*-associated missense mutation, N182K, was located upstream of the DHH/DHHA1 functional domain and led to loss of protein function. In previous studies, our group demonstrated that the loss of GdpP activity resulted in elevated levels of intracellular CDA. We also demonstrated that the loss of GdpP activity led to tolerance towards NGBs. While resistance is characterized by an increase in MIC towards antibiotics, tolerance is a phenomenon where bacteria show increased survival in the presence of antibiotics without resulting in a change in MIC [86]. Our studies hitherto revealed that altered PBP4 expression led to NGB resistance, (which is associated with a significant increase in MIC), albeit not at a very high level, while loss of GdpP function caused NGB tolerance (which is not associated with an increase in MIC). However, alterations associated with neither PBP4 (which only caused NGB resistance), nor GdpP (which only caused NGB tolerance and not resistance) could solely account for the high-level, MRSA-like NGB resistance seen in CRB. It was thus necessary to determine if the high MIC displayed by CRB was potentially attributed to alterations associated with the functioning of both PBP4 and GdpP [80]. The alterations in PBP4 and GdpP were introduced in SF8300ex to generate the triple mutant, SF8300ex Ppbp4* pbp4** Δ*gdpP* (Table 1). Interestingly, the MIC assay for the triple mutant reported a significant increase in resistance to nafcillin and oxacillin (64-fold), in addition to ceftaroline (eight-fold), when compared to the parent strain (SF8300ex). The increase in MIC for nafcillin demonstrated by the triple mutant was 64-fold higher than that for SF8300ex Δ*gdpP* and 16-fold higher than that for SF8300ex Ppbp4* pbp4**, suggesting a synergistic role of altered PBP4 and GdpP functioning. Thus, the introduction of *pbp4*-associated mutations (that led to resistance) and the deletion of *gdpP* (that resulted in tolerance), together, synergistically led to high-level NGB resistance. This synergistic effect was validated by including the *pbp4* function-defective mutation, S75A, in the triple mutant, or by complementing the triple mutant with a functional GdpP, which both resulted in the loss of resistance to NGBs. Resistance to NGBs observed in the triple mutant was comparable to that displayed by SF8300, an MRSA background strain. Moreover, in the case of ceftaroline, the MIC for the triple mutant was four-fold higher than that of the MIC for SF8300. Our results were further substantiated with the help of growth assays, which demonstrated that the triple mutant had increased survival in the presence of NGBs. These studies demonstrated that alterations associated with both PBP4 and GdpP synergistically resulted in high-level, MRSA-like NGB resistance, as represented by significantly high MIC values and increased survival in the presence of NGBs.

While the above study substantiated that both PBP4 and GdpP alterations were required for synergistic, high-level, MRSA-like NGB resistance, the mechanism by which it occurs remains elusive. GdpP alterations have been postulated to cause increased PBP4 expression; however, Bocillin-Fl assay revealed that PBP4 overexpression levels remained unchanged without or with Δ*gdpP.* Similarly, the loss of GdpP did not have any effect on the peptidoglycan cross-linking, as both the double mutant (SF8300ex Ppbp4* pbp4**) and the triple mutant had similar peptidoglycan profiles [80]. Further studies are warranted to elucidate the mechanism of synergistic action between PBP4 and GdpP.

### 5.2. PBP4’s Role in Virulence

In addition to resistance, studies have suggested that PBP4 potentially has a role in virulence. While PBP4 was identified as an important factor for bone invasion during osteomyelitis in mice [87], other studies have suggested that strains lacking PBP4 were more virulent than strains containing PBP4 [14]. Our group’s findings suggested that PBP4 overexpression led to decreased virulence in a *C. elegans* infection model [82]. Co-infection of *C. elegans* with SF8300 and SF8300 Ppbp4* demonstrated significantly decreased bacterial colonization by SF8300 Ppbp4* compared to its wild-type counterpart, suggesting that the mutant was potentially deficient in its ability to sufficiently cause virulence. Taken together, the exact role played by PBP4 in bacterial pathogenesis remains unknown and requires further investigation.

## 6. Conclusions and Future Outlooks

Our initial review discussed PBP4’s role in NGB resistance in laboratory-generated strains of *S. aureus*. However, aspects such as its clinical relevance, the effect in MRSA background, or role in virulence were unknown. Studies conducted by our group in the recent years have, as discussed above, shed more light on the roles of PBP4, which can be summarized as follows: (1) we have established PBP4 as an impactful mediator of NGB resistance in both MSSA and MRSA backgrounds and indicated that, when overexpressed, PBP4 can supplement resistance conferred by PBP2a in MRSA strains; (2) moreover, we have shown that this mechanism of resistance is consistent in hospital-associated backgrounds (such as COLnex), as well as community-associated backgrounds (such as SF8300ex); (3) we also demonstrated that *pbp4*-associated mutations (regulatory-site-associated and/or missense mutations) are detected in clinically isolated strains across the globe and can mediate NGB resistance through altered PBP4 expression and/or activity; (4) we also revealed that regulatory-site-associated mutations cause the upregulation of *pbp4* transcripts and the downregulation of *abcA* transcripts, substantiating the relevance of PBP4 overexpression in NGB resistance; (5) our findings regarding co-infection of *C. elegans* indicated that PBP4 overexpression likely resulted in decreased virulence; (6) and, more significantly, our findings uncovered the synergistic effect of altered PBP4 and GdpP functions on NGB resistance.

In addition to being the topic of our investigations, PBP4 has gained attention from several other research groups, too. (1) PBP4 has been demonstrated to be associated with proteins of the cell divisiome. It colocalizes with FtsZ [88], the essential cell division protein, and interacts with GpsB [89,90], a protein responsible for the maintenance of the cell shape; understanding its interactions with vital proteins has the potential to reveal novel targets for therapeutic development. (2) PBP4 itself has been a target of antibiotic development—small molecule screening has resulted in the identification and development of inhibitors targeting PBP4 [91,92]. Additionally, using machine learning, natural compounds with the ability to inhibit the mutant variety of PBP4 (containing the R200L mutation) have been identified [93]. The potential of using anti-infectives such as branched polyethylenimine (BPEI) [94] or daptomycin [95] in combination with NGBs is also being explored to bring about targeted therapy. (3) The prevalence of *pbp4*-associated mutations in clinical isolates of *S. aureus* is being monitored globally. Recently, whole genome sequencing and phenotypic characterization were used to survey and detect mutations associated with *pbp4* and *gdpP*, leading to functional alterations in oxacillin-resistant samples isolated from the NICU [96]. A similar surveillance study in Japan identified *pbp4* and *gdpP*-associated mutations and underscored the importance of accurate detection of MRLM isolates from MSSA and MRSA strains [97].

Together, the findings discussed in this review put forth the suggestion to consider PBP4 as a factor during clinical diagnosis and treatment of *S. aureus* infections. The identification of MRSA is done by genomic techniques such as PCR for the *mecA* gene, a gold standard test attributed due to being rapid, accurate, and cost-effective [98]. However, PCR for *mecA* is not always sufficient, as the presence of *mecC* instead of *mecA* yields a false-negative result for PCR tests specific to *mecA*. PCR for other elements of the SCC-*mec* cassette is also included during testing [62]. Phenotypic tests such as MIC determination through broth microdilution and disk tests with cefoxitin or oxacillin have proven to be rapid and accurate, due to which they are often performed in combination with PCR to differentiate MRSA and MSSA [99]. However, these tests may not be sufficient for accurate detection of MRLM or other non-classical NGB-resistant strains. MRLM strains, due to a lack of *mec* genes, are likely to be diagnosed and consequently treated as MSSA, resulting in their exposure to sub-inhibitory doses of β-lactams. Not only would this treatment be ineffective, but it could also lead to further accumulation of resistance mutations. Infections caused by MRLM strains with increased expression of PBP4 likely require treatment with increased doses of β-lactam when compared to infections caused by MSSA strains or require treatment with other classes of antibiotics. The presence of *pbp4*-associated mutations in MRSA background strains introduces further complications to pathogen diagnosis and treatment. Thus, the detection of PBP4 expression levels during infection diagnosis could potentially help lead to more specific and more effective treatment options.

While this review summarizes the current understandings of PBP4-mediated NGB resistance, there is much that remains unknown. The exact alterations brought about by the regulatory-site-associated mutations need to be identified. While it is likely that the mutations lead to alterations in binding of regulatory factors, the identification of those proteins could be beneficial for designing treatment options. Furthermore, the identification of the mechanism(s) of synergy between alterations in PBP4 and GdpP is necessary. The effect of PBP4-overexpression on other cell wall targeting antibiotics is unknown. Lastly, the effect of PBP4 overexpression on antibiotic resistance and virulence in suitable host systems should also be determined by performing *ex vivo* and *in vivo* experiments. A comprehensive understanding of the mechanisms of NGB resistance employed by PBP4, combined with development of effective diagnostic and therapeutic tools targeting PBP4, is required to curb the transmission of NGB-resistant *S. aureus* isolates.

## Figures and Tables

**Table 1:** Description of the strains discussed in this review. Mutations asssociated with the *pbp4* regulatory-site (P*pbp4*) are denoted by a single asterisk (*) whereas missense mutations associated with *pbp4*are denoted using two asterisks (**).

No.	Strain	Description
1	COL	Archaic hospital-associated MRSA strain.
2	COLnex	*mecA* excised variant of COL.
3	COLnex Δ*pbp4*	COLnex with *pbp4* deletion.
4	CRB	COLnex passaged in ceftobiprole. Contains mutations associated with *pbp4* (a 36 bp duplication 290 bp upstream of the *pbp4* start codon and missense mutations E183A, F241R) and *gdpP* (missense mutation N182K).
5	COLnex Ppbp4* (CRB)	COLnex containing regulatory-site-associated mutation detected in CRB (a 36 bp duplication 290 bp upstream of the *pbp4-* start codon).
6	COLnex pbp4** (CRB)	COLnex containing missense mutations detected in CRB (E183A, F241R).
7	COLnex Ppbp4* (CRB) pbp4** (CRB)	COLnex containing both regulatory-site-associated and missense mutations detected in CRB (A 36 bp duplication 290 bp upstream of the *pbp4* start codon and missense mutations E183A, F241R).
8	Strain 1	Clinically isolated MRLM strain. Contains T to A substitution 266 bp upstream of the *pbp4* start codon.
9	COLnex Ppbp4* (Strain 1)	COLnex containing regulatory-site-associated mutation detected in Strain 1 (T to A substitution 266 bp upstream of the *pbp4* start codon).
10	COLnex pbp4** (R200L)	COLnex containing *pbp4*-associated missense mutation (R200L) detected in a clinically isolated MRLM strain.
11	SF8300	Derivative of USA300, a community-associated MRSA strain.
12	SF8300 P*pbp4*	SF8300 containing regulatory-site-associated mutation detected in CRB (a 36 bp duplication 290 bp upstream of the *pbp4* start codon).
13	SF8300ex	*mecA* and *blaZ* excised variant of SF8300.
14	SF8300ex Δg*dpP*	SF8300ex with *gdpP* deletion.
15	SF8300ex Ppbp4* pbp4**	SF8300ex containing both regulatory-site-associated and missense mutations detected in CRB (A 36 bp duplication 290 bp upstream of the *pbp4* start codon and missense mutations E183A, F241R). Also referred to as “double mutant.”
16	SF8300ex Ppbp4* pbp4** Δg*dpP*	SF8300ex containing mutations detected in CRB, including mutations associated with *pbp4* (a 36 bp duplication 290 bp upstream of the *pbp4* start codon and missense mutations E183A, F241R) and *gdpP* (missense mutation N182K). Also referred to as “triple mutant.”

## Data Availability

No new data were created or analyzed in this study. Data sharing is not applicable to this article.
